# Supplementary material for: Modeling corticotroph deficiency with pituitary organoids supports the functional role of NFKB2 in human pituitary differentiation
Source: eLife. 2024 Nov 28;12:RP90875. doi: 10.7554/eLife.90875 (PMC11604219; doi:10.7554/eLife.90875)
Supplement: Figure 1—figure supplement 2—source data 1. [file elife-90875-fig1-figsupp2-data1.zip › Figure 1-Figure supplement 2 source data 1/Figure 1-Figure supplement 2 source data 1.pdf]

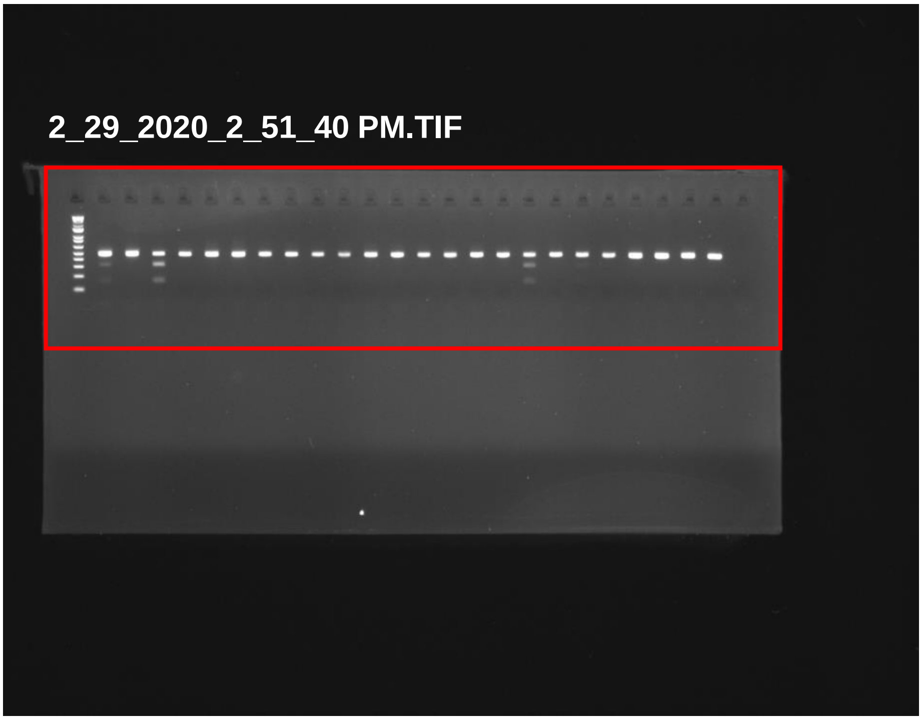

Figure 1-Figure supplement 2A Upper picture

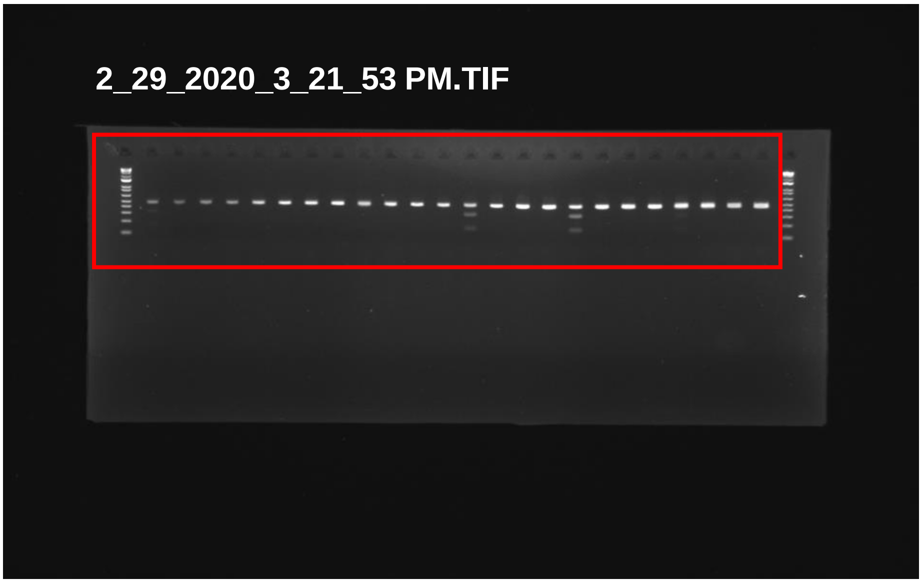

Figure 1-Figure supplement 2A Middle picture

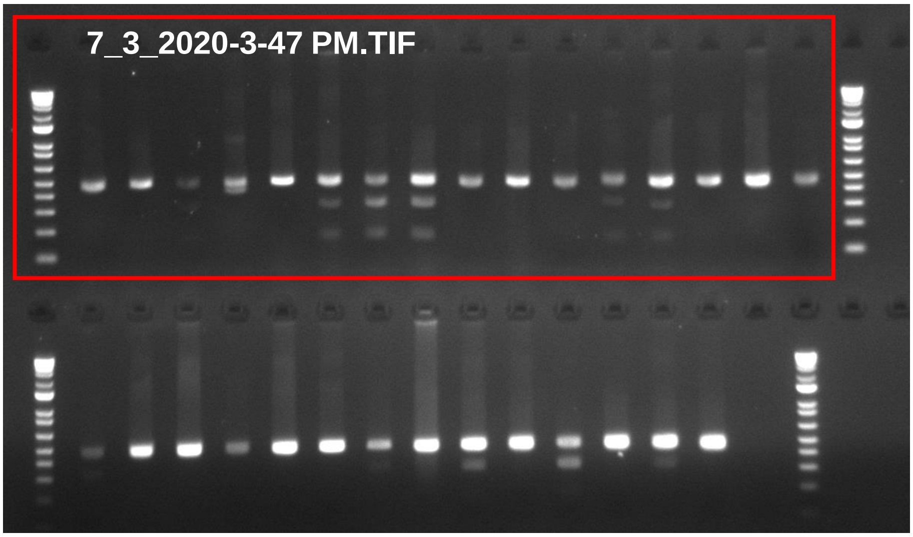

Figure 1-Figure supplement 2A Lower picture

Figure 1-Figure supplement 2 source data 1: Original pictures of gels corresponding to Figure 1-Figure supplement 2A. Pictures were cropped (red frame) and the LUT was inverted
