# Supplementary figures and images for: Modeling corticotroph deficiency with pituitary organoids supports the functional role of NFKB2 in human pituitary differentiation
Source: eLife. 2024 Nov 28;12:RP90875. doi: 10.7554/eLife.90875 (PMC11604219; doi:10.7554/eLife.90875)

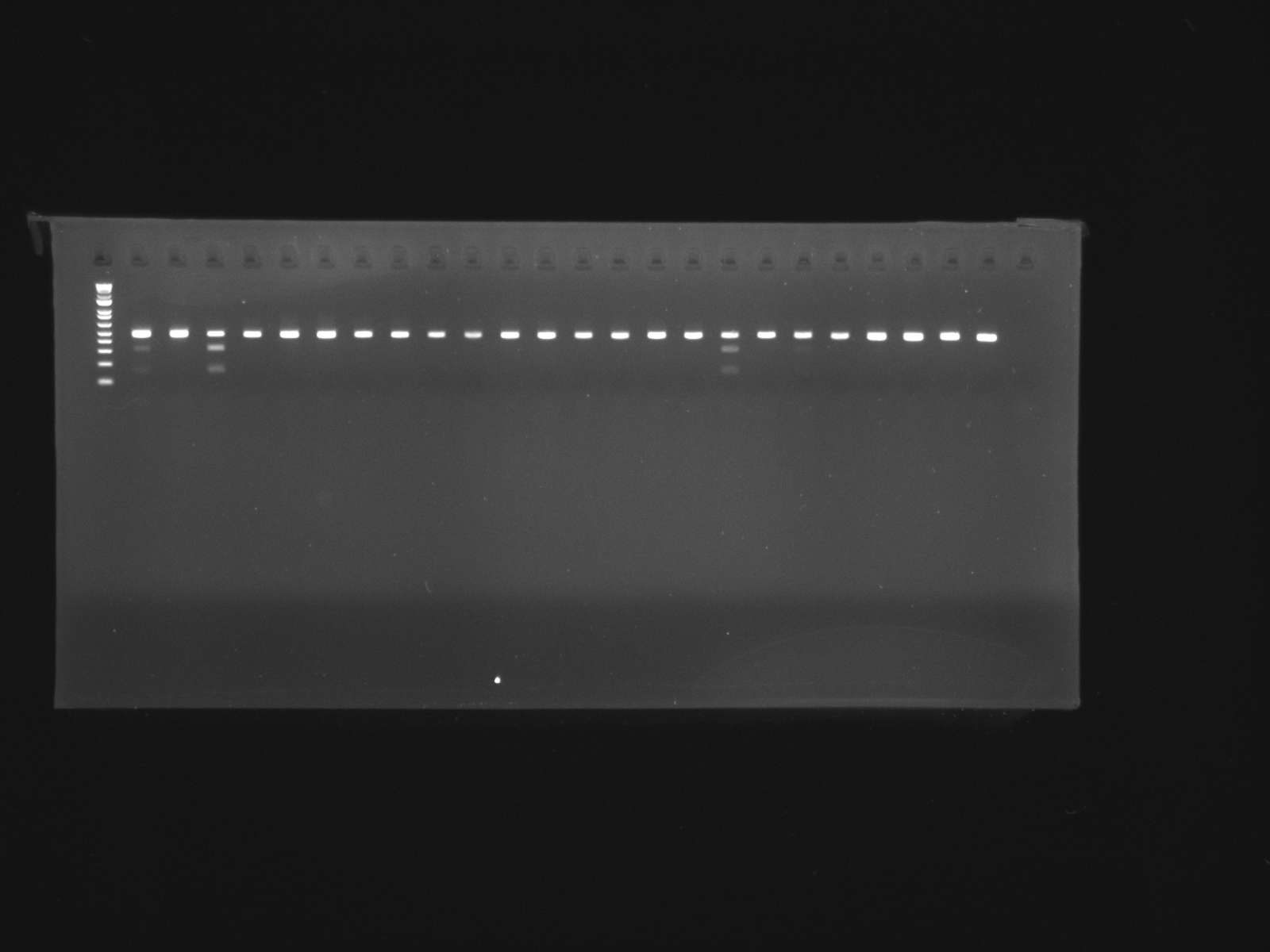

Supplement: Figure 1—figure supplement 2—source data 2. [file elife-90875-fig1-figsupp2-data2.zip › Figure 1-Figure supplement 2 source data 2/2_29_2020_2_51_40 PM.Tif]

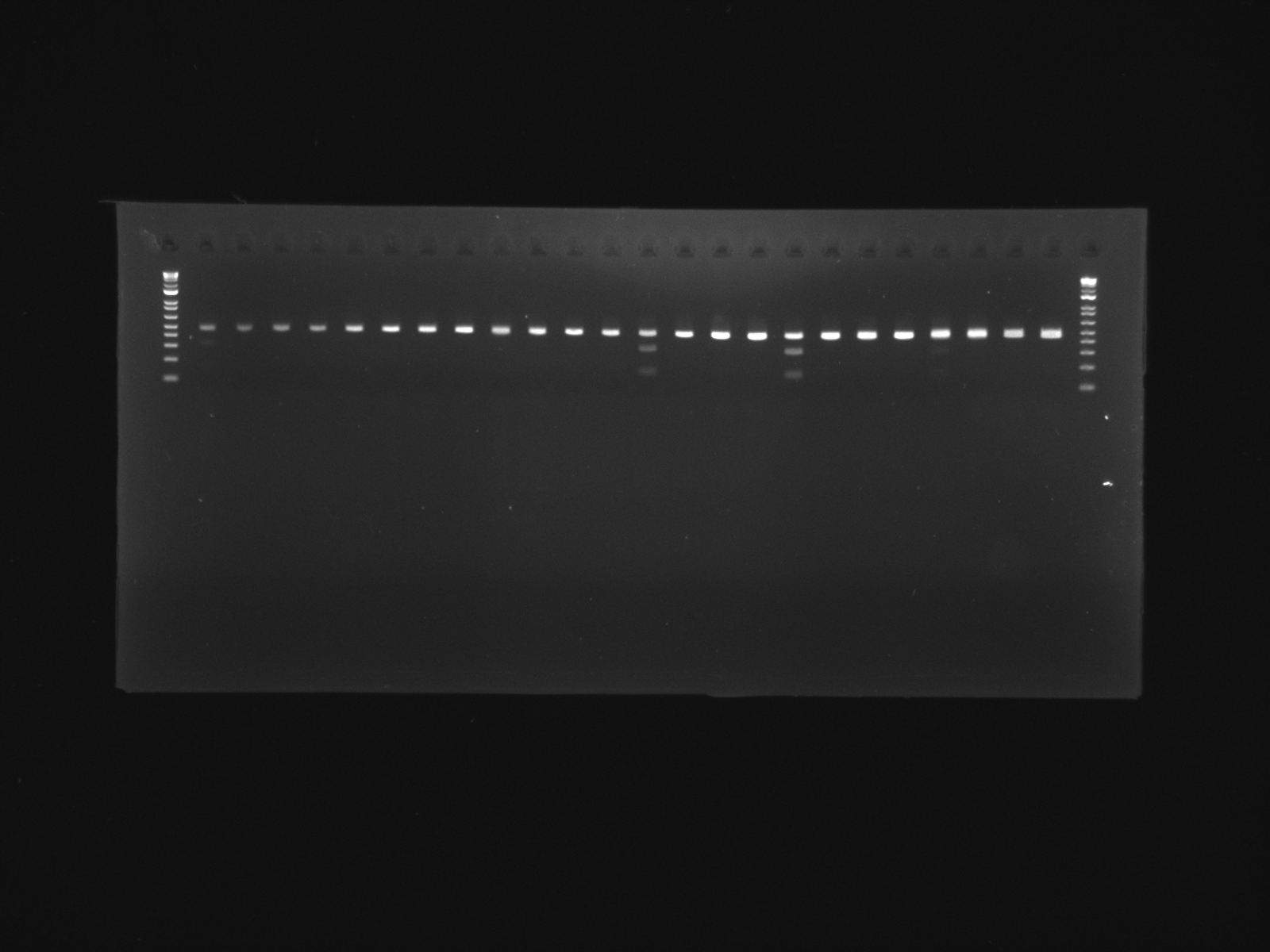

Supplement: Figure 1—figure supplement 2—source data 2. [file elife-90875-fig1-figsupp2-data2.zip › Figure 1-Figure supplement 2 source data 2/2_29_2020_3_21_53 PM.Tif]

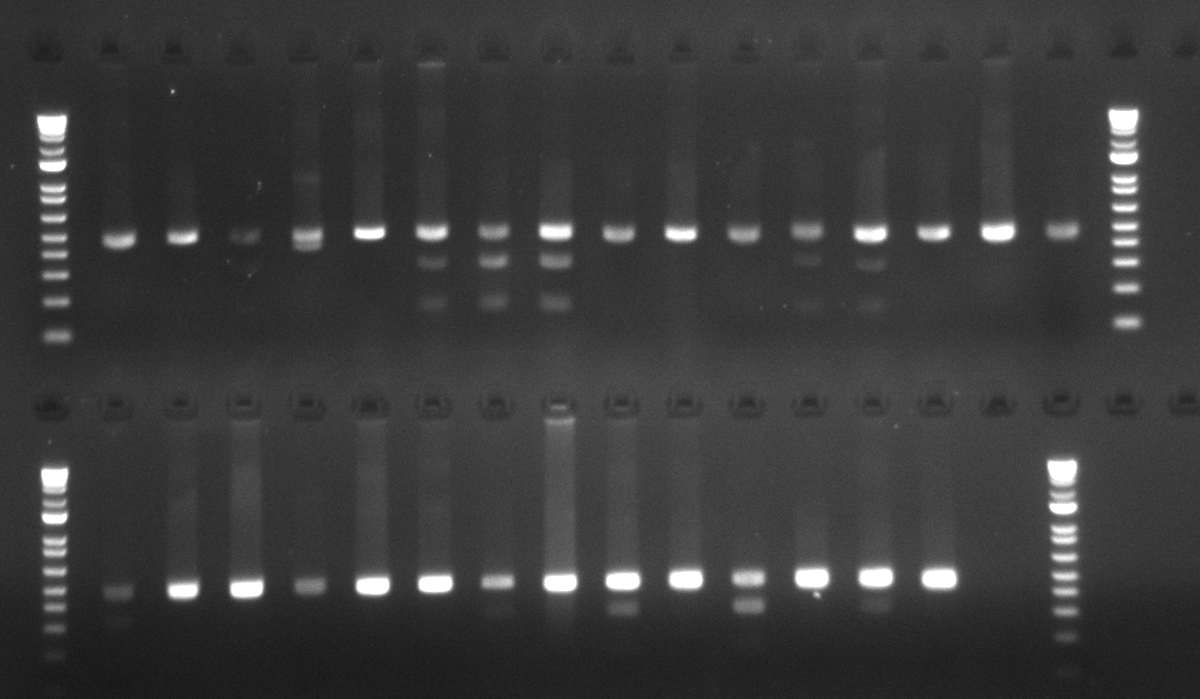

Supplement: Figure 1—figure supplement 2—source data 2. [file elife-90875-fig1-figsupp2-data2.zip › Figure 1-Figure supplement 2 source data 2/7_3_2020-3-47 PM.tif]

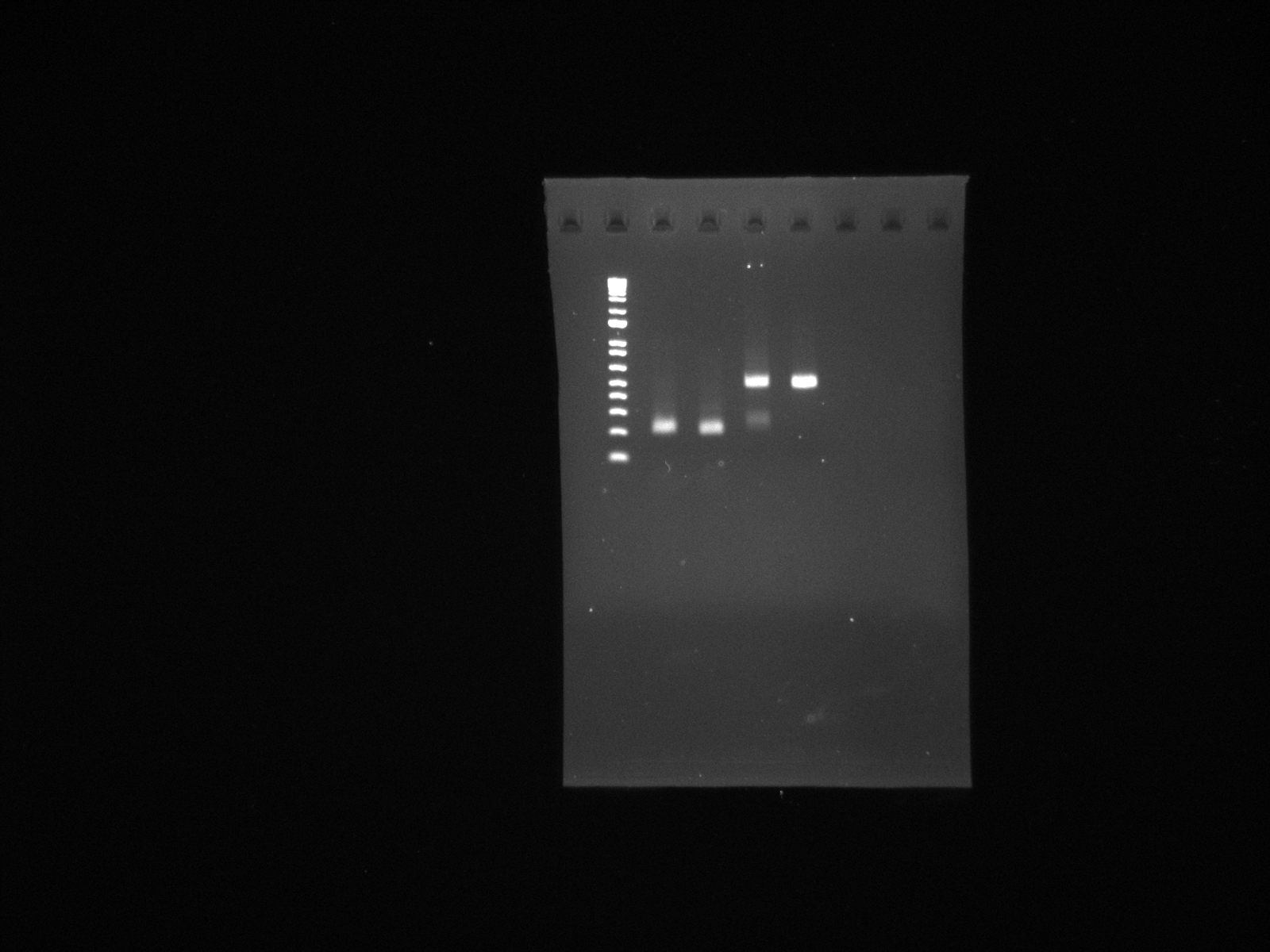

Supplement: Figure 5—figure supplement 1—source data 2. [file elife-90875-fig5-figsupp1-data2.zip › Figure 5-Figure supplement 2 source data 2/1_29_2020_2_35_06 PM.Tif]

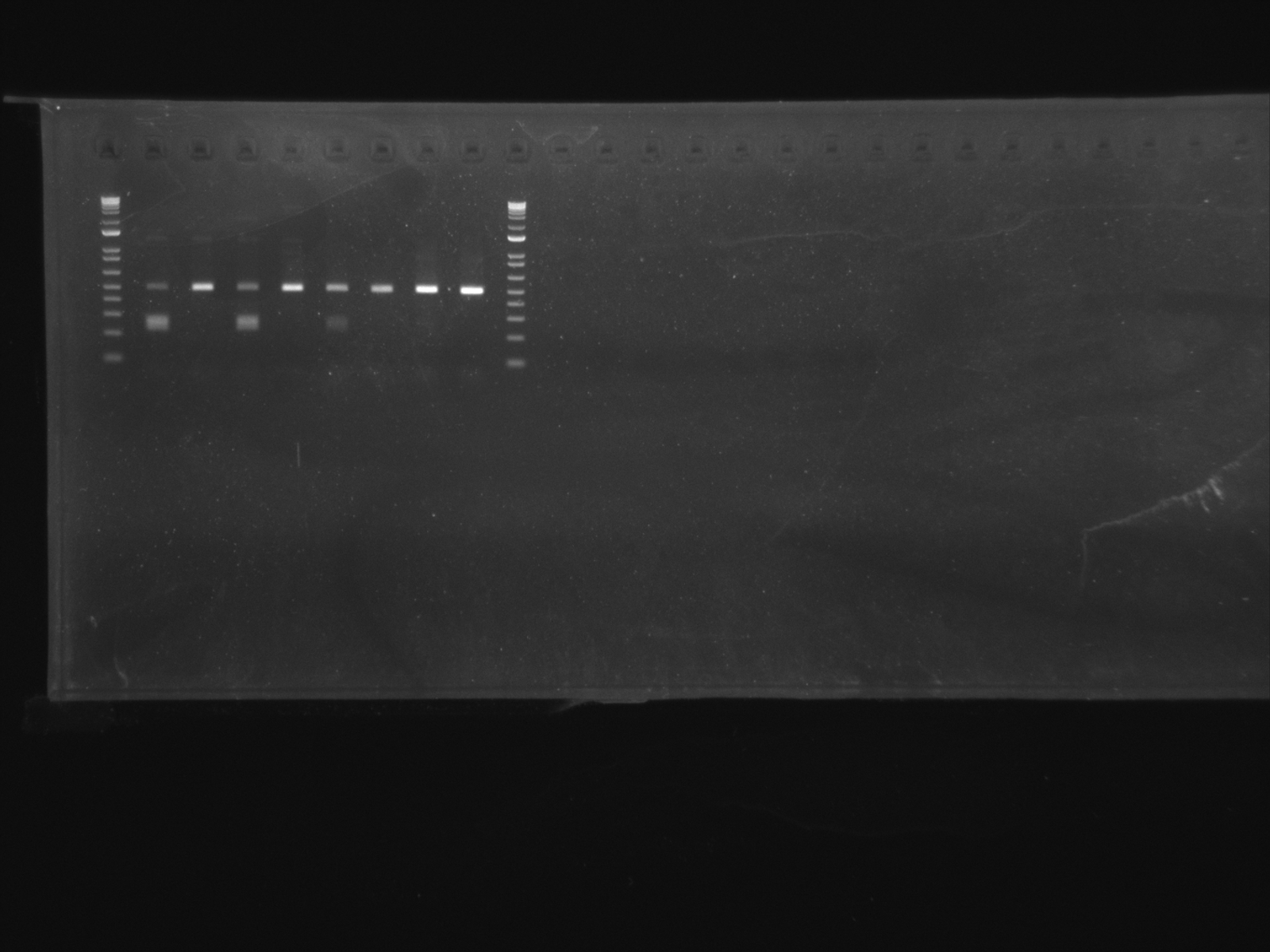

Supplement: Figure 5—figure supplement 1—source data 2. [file elife-90875-fig5-figsupp1-data2.zip › Figure 5-Figure supplement 2 source data 2/2_19_2020_3_32_04 PM.Tif]
