# Supplementary material for: Modeling corticotroph deficiency with pituitary organoids supports the functional role of NFKB2 in human pituitary differentiation
Source: eLife. 2024 Nov 28;12:RP90875. doi: 10.7554/eLife.90875 (PMC11604219; doi:10.7554/eLife.90875)
Supplement: Figure 5—figure supplement 1—source data 1. [file elife-90875-fig5-figsupp1-data1.zip › Figure 5-Figure supplement 1 source data 1/Figure 5-Figure supplement 1 source data 1.pdf]

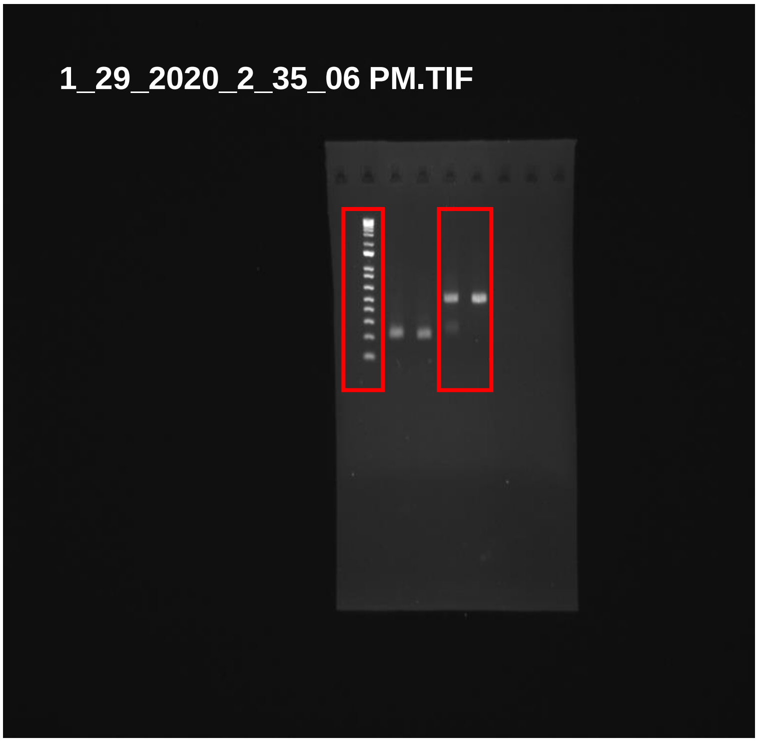

Figure 5-Figure supplement 1A

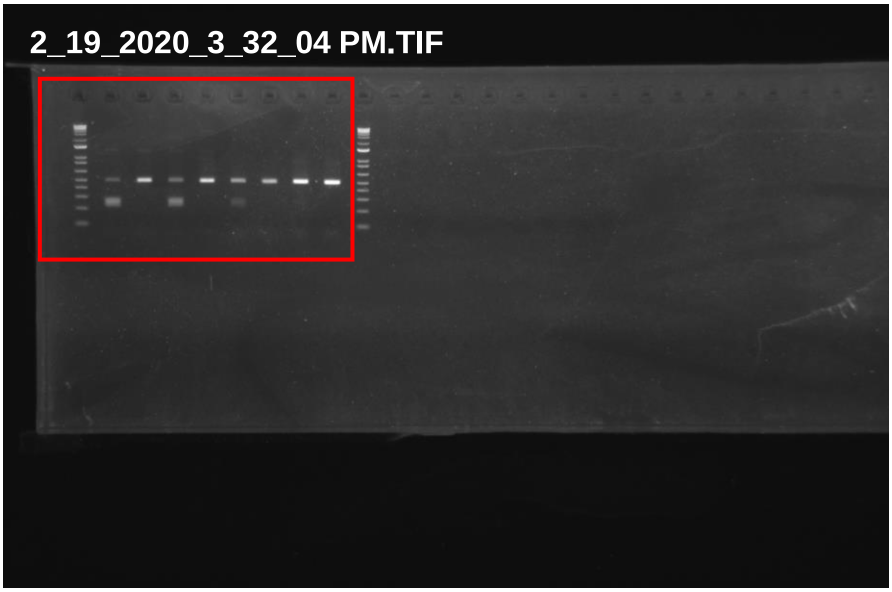

Figure 5-Figure supplement 1B

Figure 5-Figure supplement 1 source data 1: Original pictures of gels corresponding to Figure 5-Figure supplement 1. Pictures were cropped (red frame) and the LUT was inverted
