# Supplementary material for: Modeling corticotroph deficiency with pituitary organoids supports the functional role of NFKB2 in human pituitary differentiation
Source: eLife. 2024 Nov 28;12:RP90875. doi: 10.7554/eLife.90875 (PMC11604219; doi:10.7554/eLife.90875)
Supplement: Supplementary file 1. — (a) hiPSC control line used in the study. (b) List of oligonucleotides used for CRISPR/Cas9 experiments to target TBX19K146R. (c) List of oligonucleotides used for CRISPR/Cas9 experiments to target NFKB2D865G. (d) List of primers used for PCR analyses, CAPS assay, and Sanger Sequencing. (e) PCR settings for TBX19. (f) PCR settings for NFKB2. (g) List of primers used for qRT-PCR. (h) List of primary antibodies used for immunostainings. [file elife-90875-supp1.docx]

**Supplementary file 1**

| **Line** | **Sex** | **Age** | **Derivation** | **Reprogramming method** | **Reprogramming factors** |
| --- | --- | --- | --- | --- | --- |
| 10742L | Female | 82 | Fibroblast | Nucleofection | OCT4, SOX2, KLF4, c-MYC, SH-P53 |

**Supplementary file 1a: hiPSC control line used in the study**

| **Nomenclature used in this paper** | **Sequence** |
| --- | --- |
| sgRNA | AAGCTGACCAACAAGCTCAA |
| sgRNA oligo up | 5’-CACCGAAGCTGACCAACAAGCTCAA-3’ |
| sgRNA oligo down | 5’-AAACTTGAGCTTGTTGGTCAGCTTC-3’ |
| ssODN | TGGATGAAAGCTCCCATCTCCTTCAG  CAAAGTGAGGCTGACCAACAAGTTaAA  TGGAGGCGGGCAGGTACGAATGAGG  CGGGCAGGCCTGGCCACCCGCT |

**Supplementary file 1b: List of oligonucleotides used for CRISPR/Cas9 experiments to target *TBX19^K146R^***

| **Nomenclature used in this paper** | **Sequence** |
| --- | --- |
| sgRNA | GTGAAGGAAGACAGTGCGTA |
| sgRNA oligo up | 5’-CACCGTGAAGGAAGACAGTGCGTA-3’ |
| sgRNA oligo down | 5’-AAACTACGCACTGTCTTCCTTCAC-3’ |
| ssODN | TCCCATTCCTGTCCCCATTTACCCCC  AGCAGAGGTGAAGGAAGGCAGTGCC  TACGGGAGCCAGTCAGTGGAGCAGG  AGGCAGAGAAGCTGGGCCCACCCC |

**Supplementary file 1c: List of oligonucleotides used for CRISPR/Cas9 experiments to target *NFKB2^D865G^***

| **Gene** | **Forward** | **Reverse** |
| --- | --- | --- |
| *NFKB2* | CCCTAACCATGACTCAGACCTCA | CCTCCCCTTCCCATGAGAATCC |
| *TBX19* | CCCCTGGACAAGGTGAGAGTT | GACTCCCGGGAATAATTGGCTTC |

**Supplementary file 1d: List of primers used for PCR analyses, CAPS assay and Sanger Sequencing**

|  | **Time** | **Temperature** | **Cycle** |
| --- | --- | --- | --- |
| **Initial denaturation** | 1 min | 94°C |  |
| **Denaturation** | 10 sec | 98°C | 40 cycles |
| **Hybridization** | 30 sec | 62.5°C |  |
| **Elongation** | 30 sec | 68°C |  |
| **Final elongation** | 1 min | 68°C |  |

**Supplementary file 1e: PCR process for *TBX19***

|  | **Time** | **Temperature** | **Cycle** |
| --- | --- | --- | --- |
| **Initial denaturation** | 1 min | 98°C |  |
| **Denaturation** | 10 sec | 98°C | 40 cycles |
| **Hybridization** | 10 sec | 66°C |  |
| **Elongation** | 30 sec | 68°C |  |
| **Final elongation** | 1 min | 68°C |  |

**Supplementary file 1f: PCR process for *NFKB2***

| **Gene** | **Forward** | **Reverse** |
| --- | --- | --- |
| *ACTB* | CGGGAAATCGTGCGTGACATTAAG | GTAGTTTCGTGGATGCCACAGGA |
| *GAPDH* | CGGAGTCAACGGATTTGGTCGTAT | CAGCATCGCCCCACTTGATTTTG |
| *TUBB* | TGAGGGAAATCGTGCACATCCA | CCAAAAGGACCTGAGCGAACAGA |
| *BMP4* | ACCTCGGCCAAGTAACGGTAGT | TCCGGACTACATGCGGGATCTTTA |
| *FGF10* | TGTGCGGAGCTACAATCACCTT | CAGGATGCTGTACGGGCAGTT |
| *FGF8* | ACACCTTTGGAAGCAGAGTTCGAG | TGAAGACGCAGTCCTTGCCTTT |
| *HESX1* | CGCTCAGCTCGGGGAAAACAAA | ACCACGCTAGGGAATGAAATCCCA |
| *LHX3* | TGGTGCAGGTTTGGTTCCAGA | ATTTCCGCCAAGGAAGGCTCAT |
| *NEUROD4* | CCAGTGACCGAGAGTCTGGA | ACCTCATTTTGGGAGCCCAGA |
| *NR4A2* | AGGTTCCAGGCGAACCCTGACT | AGGTCTGCGAAGCCAGGGATCT |
| *PCSK1* | CAACTATGATCCAGAGGCTAGC | TCTGGTCCCGTGTTTGTTC |
| *PITX1* | GGCAACGTACGCACTTCACAA | TGCACTAGGCCGCTGAACT |
| *POMC* | CCCCTACAGGATGGAGCACTT | GATGGCGTTTTTGAACAGCGT |
| *PROP1* | GCAGTTGGAACAGCTGGAGTCA | AAGCAGTGGACTCTGGCAAGAA |
| *TBX19a* | TGAGAGCCAGCATGTGACCTAT | TGTGCATGTACGCAGAAGGGT |
| *TBX19b* | TGGCAGACGGATGTTTCCAGTC | CACCCATTCCCCGTTGACGTAC |
| *ZBTB20* | AACGGAAGAAACCCAAGACAGCT | AGCTTCAAAGTTCAGGCAGGGA |

**Supplementary file 1g: List of primers used for qRT-PCR**

| **Antibody** | **Host species** | **Company and catalog number** | **Dilution** |
| --- | --- | --- | --- |
| Anti-NFKB2 | Rabbit | Sigma, HPA008422  RRID:AB_1854434 | 1/500 |
| Anti-LHX3 | Mouse | DSHB, 67.4E12  RRID:AB_2135805 | 1/500 |
| Anti-TBX19 | Rabbit | Sigma, HPA072686  RRID:AB_2732209 | 1/300 |
| Anti-ACTH | Guinea Pig | NIDDK | 1/2000 |
| Anti-E-cadherin | Rat | Millipore, MABT26  RRID:AB_10807576 | 1/500 |
| Anti-NKX2.1 | Mouse | Millipore, MAB5460  RRID:AB_571072 | 1/500 |
| Anti-PITX1 | Rabbit | Sigma, HPA008743  RRID:AB_1855413 | 1/500 |

**Supplementary file 1h: List of primary antibodies used for immunostaining**
